# Comparing Written Versus Pictorial Asthma Action Plans to Improve Asthma Management and Health Outcomes Among Children and Adolescents: Protocol of a Pilot and Feasibility Randomized Controlled Trial

**DOI:** 10.2196/11733

**Published:** 2019-06-17

**Authors:** Lisa Hynes, Kristine Durkin, Desireé N Williford, Hope Smith, David Skoner, Christa Lilly, Viral Dilip Kothari, Jenny Mc Sharry, Christina L Duncan

**Affiliations:** 1 Department of Psychology West Virginia University Morgantown, WV United States; 2 Department of Pediatrics School of Medicine West Virginia University Morgantown, WV United States; 3 Department of Biostatistics School of Public Health West Virginia University Morgantown, WV United States; 4 School of Psychology National University of Ireland, Galway Galway Ireland

**Keywords:** asthma, child, adolescent, education, self-management

## Abstract

**Background:**

Asthma is an important focus for pediatric health research as management of asthma symptoms is a significant challenge, and morbidity and mortality among youths with asthma remain prevalent. Treatment guidelines for asthma recommend a written asthma action plan (WAAP) that summarizes individualized instructions for daily medication use. However, WAAPs are typically written at a seventh- to ninth-grade reading level, which can be a barrier to young people in understanding their treatment, having confidence in using a WAAP, and engaging with asthma education.

**Objective:**

Utilizing a feasibility and pilot randomized controlled trial (RCT) design, the objective of the *Take Action for Asthma Control* study is to test a symptom-based, computer-generated pictorial asthma action plan (PAAP) in comparison with a standard WAAP and assess the feasibility and acceptability of the asthma action plan (AAP) intervention and study procedures. The study has 3 aims: (1) estimate the effect sizes of PAAPs compared with WAAPs on outcomes (eg, AAP knowledge and medication adherence), (2) evaluate feasibility and acceptability of AAP intervention and RCT procedures from the perspectives of key stakeholders, and (3) establish whether parent and youth literacy levels are associated with treatment outcomes.

**Methods:**

This feasibility and pilot RCT is a block randomized, 2-arm, parallel-group clinical trial, lasting 6 months in duration. At baseline, participants will be randomly assigned to receive a PAAP or WAAP generated for them and reviewed with them by their asthma physician. Study procedures will take place over 4 separate time points: a baseline clinic appointment, 1-month telephone follow-up, and 3- and 6-month clinic-based follow-ups. At each time point, data will be collected related to the main outcomes: AAP knowledge, AAP satisfaction, asthma control, pulmonary function, and adherence to daily asthma medication. A sample size of up to 60 participants (aged 8-17 years) will be recruited. Feasibility and acceptability data will be collected via one-to-one qualitative interviews with providers involved in the study and a subgroup of families that participate in the study.

**Results:**

Recruitment and data collection began in May 2017 and were completed in October 2018.

**Conclusions:**

This pilot and feasibility study will test the potential efficacy, feasibility, and acceptability of an AAP intervention and study procedures. The findings will inform the design and delivery of a future definitive trial to assess the efficacy of PAAPs versus WAAPs in supporting asthma self-management among children and adolescents.

**International Registered Report Identifier (IRRID):**

DERR1-10.2196/11733

## Introduction

### Background

Asthma affects over 10 million children and adolescents in the United States [1]. Suboptimal asthma management is associated with morbidity (eg, school absences and asthma attacks) [2] and even mortality [1]. Effective asthma management requires that both preventive and rescue medications are taken at the appropriate times and a clear understanding of when to seek emergency care. Current best practices for asthma management include administration of an asthma action plan (AAP), and providing specific, individualized instruction to people with asthma and caregivers regarding their daily treatment regimen [3]. The use of written asthma action plans (WAAPs) can be associated with a number of improved health outcomes, including fewer hospitalizations and improved adherence to medications [4,5]. However, possible literacy deficits [6], lack of self-efficacy in relation to using an AAP [7], and insufficient asthma education [8,9] are important and significant barriers to both providers and families accepting and using AAPs effectively.

Given that most WAAPs consist of densely presented text typically written at a seventh- to ninth-grade reading level [10], their utility could be reduced for people with literacy concerns. Low parental literacy, which is often reported in rural populations [11-13], is associated with significantly poorer outcomes in children with asthma, such as an increased number of emergency department visits and school absences [13]. Moreover, although children as young as 9 years old are principally responsible for their asthma medication use [14], young people may not be engaged in asthma education during clinic visits, negatively impacting the development of their asthma literacy and self-management skills [8]. A recent report found that in the United States, only 50.8% of young people under the age of 18 years receive an AAP [7]. Concerns related to the accessibility of AAPs, the literacy levels, and the responsibility placed on young people to manage their asthma provide a rationale for developing a simplified and individualized AAP that can facilitate engaging young people and families in asthma education and in daily asthma management.

Visual tools for communicating health-related information (eg, details about a diagnosis and instructions for treatment) can improve comprehension, satisfaction with information, self-management, and provider-patient engagement [15-17]. Recent AAP research has integrated the use of images with different amounts of supporting text [18,19] or mobile apps [20] to communicate asthma treatment plans. However, the focus of this AAP research has been to develop pictorial asthma action plans (PAAPs) and electronic AAPs [21,22] and to evaluate the feasibility and acceptability of their use [20,23]. Existing research has focused on the perspectives and behaviors of parents with little integration of the perspectives of young people [19]. The impact of PAAPs or electronic AAPs on the comprehension of asthma treatment among children has not been investigated to date, nor has the impact of PAAPs on parent or youth asthma management behavior.

### Study Objective

This protocol describes the final phase in a 2-phase study, called the *Take Action for Asthma Control* (TAAC) study, which aims to assess the feasibility, acceptability, and preliminary efficacy of a symptom-based, computer-generated PAAP for supporting pediatric asthma management relative to a standard text-based WAAP. In the first phase, PAAP software was developed in consultation with young people with asthma, their parents and asthma providers, and a health technology company [24]. This software enables providers to generate simplified and tailored PAAPs that reflect the patient’s asthma treatment regimen. PAAPs primarily comprise graphics and images, with minimal words or phrases.

Assessing feasibility and acceptability of interventions in health care settings will ensure that important factors related to intervention design and study procedures are explored and optimized before implementing a definitive randomized controlled trial (RCT) [25]. Moreover, this focus during the intervention development and pilot testing phases is now generally considered a prerequisite for successful implementation of interventions into clinical practice [26]. On the basis of guidance for conducting pilot and feasibility studies [27,25], the approach to data collection in this study was designed to answer pertinent questions ahead of a future definitive trial. Thus, feasibility-related questions are concerned with understanding whether or not a future trial and routine use of PAAPs in practice may be possible. For example, fit of the study activities within the flow of asthma clinics is one of our feasibility outcomes. Acceptability-related questions will address the asthma education preferences of providers and families, and the appeal and usability of PAAPs from the perspectives of families are among the acceptability outcomes in this study.

A mixed-methods approach to data collection and analysis will be used based on the current guidelines for the development of effective behavior change interventions [28]. Using a pilot RCT design, the aims of this study are threefold: (1) assess initial evidence for the efficacy of PAAPs in comparison with WAAPs for improving pediatric asthma management and outcomes (ie, child and caregiver knowledge of treatment plan, AAP satisfaction, adherence to daily controller medication, symptom control, and lung function), resulting in robust effect size estimates ahead of a future definitive RCT; (2) evaluate the feasibility and acceptability of the PAAP software, PAAPs, and study procedures; and (3) identify whether parent health literacy and youth literacy levels are associated with outcomes.

## Methods

The study protocol, personnel, and materials have been reviewed and approved by the Institutional Review Board at West Virginia University (WVU).

### Study Design and Sample

This study is taking place in subspecialty clinics (asthma/allergy/pulmonology) across 3 locations affiliated with WVU Medicine, Department of Pediatrics. A sample of 60 children and adolescents with asthma (aged 8-17 years) and parents or caregivers or legal guardian (hereon referred to as *parent*) will be recruited. Participants will be block randomized (age: 8-12 and 13-17 years; physician-determined asthma severity: mild or moderate to severe) to 1 of the 2 groups: a PAAP group or a WAAP group. Random group assignment is identified through selection of an envelope from sets of envelopes arranged into the randomization blocks. Group assignment is concealed until the family has completed the informed consent process. Data will be collected at baseline (enrollment and intervention), with follow-up 1, 3, and 6 months later.

The inclusion criteria for the participants are as follows: (1) aged 8-17 years, (2) have a clinical diagnosis of persistent asthma, (3) have a prescription for a daily controller inhaler compatible with an adherence monitoring electronic sensor (ie, Qvar HFA (TEVA), Dulera HFA (Merck), Advair [HFA and Diskus] (GSK), or Flovent [HFA and Diskus] (GSK), (4) have never received an AAP in the past, and (5) do not have a disability or cognitive impairment that would prevent them from completing the study procedures. Eligible participants must also match at least one of the following supplemental criteria: (1) newly diagnosed with persistent asthma, (2) asthma control is suboptimal (eg, child is using his or her rescue inhaler often), or (3) physician plans to make a change to the patient’s asthma treatment plan.

Potentially eligible families will be identified through 2 pathways: (1) clinic staff will identify potential participants through the electronic medical record (EMR) system and share clinic appointment details with the research team or (2) through a filter based on eligibility criteria applied to EMR lists associated with clinics. Families identified this way will receive a brief email message through the EMR system, introducing them to the study and providing contact details for the research team. Families who do not have an active email account as part of the EMR system will receive a phone call. Interested families who are not already receiving care in one of the study’s allergy or asthma specialty clinics can be referred by their physician if a referral is deemed appropriate.

A research team member will call all families in advance of their clinic appointment to introduce or reintroduce the study so that they can plan to have enough time to stay to complete the baseline appointment, which lasts approximately 90 min.

### Study Intervention

The TAAC intervention involves an individually tailored AAP (WAAP or PAAP) summarizing each participant’s asthma treatment plan. On the basis of the National Health, Lung & Blood Institute (NHLBI) guidelines for managing persistent asthma [3] and the patient’s prescribed asthma treatment, physicians will populate AAPs according to 3 colored zones that describe treatment instructions in response to asthma symptoms characteristic of each zone: (1) green zone comprises daily management options when no symptoms are present; (2) yellow zone presents options for managing distressing asthma symptoms, including wheezing and chest tightness; and (3) red zone outlines options for managing highly distressing, even life-threatening asthma symptoms, such as severe shortness of breath. PAAPs will be generated using the PAAP software and printed onto a single page with a color printer, whereas the NHLBI WAAP template [29] will be completed by hand and photocopied for participants. Multiple copies of the AAP will be given to each family, and families will be encouraged to place the copies in convenient locations (eg, refrigerator) for prompting asthma management. The same individually tailored asthma management guidance (via AAP) is delivered to participants, regardless of the assigned intervention group. To ensure AAP review sessions are uniform across participants, physicians are encouraged to maintain consistency in reviewing details of asthma care plans, regardless of participant group assignment, thereby delivering sessions that are equivalent in length. All AAP review sessions are audio recorded, and 20% (approximately 12 recordings) of them will be randomly selected for evaluation by an independent researcher to assess intervention fidelity and uniformity across groups.

**Figure 1:**
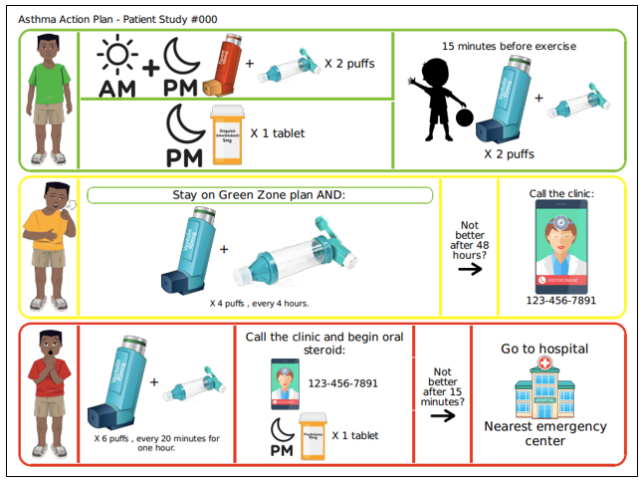
Example of personalized pictorial asthma action plan.

The PAAP software is designed to enable providers to quickly and easily generate a PAAP that is tailored to the characteristics (ie, gender and ethnicity) and asthma treatment plans (eg, daily controller inhaler type) of each participant. Physicians will generate the PAAP by responding to questions organized by zone (green, yellow, and red), and each question is followed by a drop-down menu of options. PAAPs comprise 3 horizontal banners for the green, yellow, and red zone treatment instructions (see Figure 1). Each zone begins with an avatar representing the particular child (ie, gender and race and ethnicity) and provides a visual cue for the level of distress and types of symptoms associated with each zone. A child’s favorite sport or physical activity is incorporated into the PAAP to communicate a pre-exercise prescription, if relevant. PAAPs contain various images (eg, inhalers, a spacer, and a cell phone) and symbols (eg, sun to denote the time of day) for prompts as well as a small amount of basic text and numerical information to communicate treatment instructions. The PAAP software program was designed so that it does not have the capability to store any Protected Health Information data related to individual participants.

Physician training to generate AAPs using the PAAP software will occur over a 30-minute session with the project coordinator. During these sessions, a written step-by-step guide will be provided to the physicians and they will be introduced to the software, several sample PAAPs will be generated, and issues or questions will be discussed.

The NHLBI WAAP template [30] was chosen because the NHLBI is the principal source of up-to-date and evidence-based guidelines related to asthma management in the United States. The treatment options available to providers in the PAAP software were selected based on the NHLBI guidelines [3]. In addition, the horizontal format of the NHLBI WAAP mirrors the format of the PAAP. The WAAP template enables providers to communicate the same information as that provided through the PAAP software. However, the WAAP consists of text and numerical content only, with no images included.

#### Intervention Components

The TAAC intervention comprises 5 behavior change techniques (BCTs), as defined in the BCT Taxonomy version 1 [31]. Table 1 lists the BCTs that are delivered to participants in both the PAAP and WAAP groups, the definition of each BCT according to the BCT Taxonomy, and the operationalization of each BCT in the intervention.

**Table 1:** Behavior change techniques within the Take Action for Asthma Control intervention (pictorial asthma action plan and written asthma action plan).

Behavior change techniques	Definition	Operationalization
Action planning	To prompt detailed planning of performance of the behavior (must include at least one of the following: context, frequency, duration, and intensity)	The AAP^a^ provides step-by step details of prescription for asthma medications (eg, 2 puffs twice a day) and help-seeking steps (eg, call physician).
Habit formation	To prompt rehearsal and repetition of the behavior in the same context repeatedly so that the context elicits the behavior	The AAP prompts taking medication in the morning and evening, before exercise (green zone), or in response to certain symptoms in the yellow and red zones. It also prompts to call the clinic or hospital or 911 in response to lack of symptom improvement.
Adding objects to the environment	To add objects to the environment to facilitate performance of the behavior (more than an information booklet)	The intervention adds an AAP to the family’s home environment. Families are encouraged to place the AAP somewhere easily visible.
Goal setting (behavior)	To set or agree on a goal defined in terms of the behavior to be achieved	The AAP provides step-by step details of prescription for asthma medication and help-seeking steps to follow for daily asthma management.
Credible source	To present verbal or visual communication from a credible source in favor of or against the behavior	In both the written asthma action plan and pictorial asthma action plan groups, the physician reviews the AAP during the clinic appointment.

^a^AAP: asthma action plan.

### Procedure

#### Baseline Visit

Physicians will assess families for study eligibility during their consultation and invite a researcher to discuss the study further with interested families. Families who agree to participate will provide informed consent (parent) and assent (child) and be randomized to the PAAP or WAAP groups. Group assignment will then be communicated to the physician, who will generate a WAAP or a PAAP and deliver the AAP review session with the family.

Following the AAP review session, the participant and parent will separately complete brief structured interviews to assess their comprehension of the new AAP. Participants and parents will complete a number of questionnaires (see Table 2), and questions and response options will be read aloud to individuals, if preferred. Trained researchers will also administer a standardized measure of reading comprehension and a pulmonary function test with the participant. All study documents, including the PAAP and WAAP, will be coded with the participant’s study ID number to omit identifying information.

**Table 2:** Summary of study appointment procedures.

Appointment information	Baseline	1 month	3 month	6 month
Location	Clinic	Telephone	Clinic	Clinic
Duration (min)	90	30	45	60
Gift card value	US $30	US $20	US $30	US $45
Participant information	✓^a^	—^b^	—	—
Asthma Control Test	✓	✓	✓	✓
Spirometry	✓	—	✓	✓
Reading comprehension and health literacy	✓	—	—	—
AAP^c^ knowledge	✓	—	✓	✓
AAP satisfaction	—	✓	—	✓
Daily controller adherence^d^	✓	✓	✓	✓

^a^Indicates time points at which data were collected.

^b^Indicates time points at which data were not collected in relation to a variable, eg, spirometry was not measured at 1 month follow-up as that appointment was conducted by phone.

^c^AAP: asthma action plan.

^d^Daily controller inhaler adherence is monitored continuously through the sensor attached to each participant’s inhaler. Syncing of adherence data with the sensor dashboard will be checked at 4 time points, but the data will only be discussed with families at the 6-month follow-up appointment.

To enable the collection of objective adherence data for daily controller medication, a small electronic sensor will be fitted to the participant’s inhaler. The sensor is connected to an app that is downloaded to the participant’s or their parent’s cell phone or to a device called a hub that can be plugged in at the family’s home, with individual participant data transferred to a Web-based dashboard that will be monitored by the research team. A complimentary canister or diskus (circular, rather than cylindrical inhaler device) of their daily controller inhaler will be provided to each participant to allow the research team to fit the sensor onto the inhaler and to show the family how this is done for refill medication.

#### Follow-Up Visits

Table 2 summarizes the procedure and measures at each of the 1-, 3-, and 6-month follow-up visits. In addition to the procedures outlined in Table 2, during the final study visit, the data gathered by the adherence sensor will be discussed with the family. Sensors and hubs will be collected from families, and each participant will be entered into a lottery for 1 of 6 US $50 gift cards if the sensor is in working order. See Table 2 for participant payment information. Finally, families will be invited to take part in a qualitative interview to provide feedback on being part of the study and on their AAP.

### Measures

To address aims 1 and 3 of this study, validated measures of health literacy (Short Test of Functional Health Literacy in Adults [S-TOFHLA]) and numeracy (Asthma Numeracy Questionnaire [ANQ]), reading comprehension (Wechsler Individual Achievement Test-III [WIAT-III]), and asthma symptom control (Asthma Control Test [ACT]) will be used. Details of the quantitative measurement tools that will be used are summarized in Table 3. AAP knowledge and AAP satisfaction will be assessed using a participant and parent structured interview developed for this study. Asthma symptom control and adherence to daily controller inhaler will be measured using objective methods, as described below.

**Table 3:** Overview of quantitative measures.

Measure			Length	Validation status	Cronbach alpha according to previous research	Response options	Range possible	Respondents
**Literacy assessments**								
	Short Test of Functional Health Literacy in Adults		36 items	Validated for use with young people and adults aged 13+	Youth=.90-.92; adults=.97	Fill in the blanks to complete sentences	0-36	Parents and participants aged 13-17 years
	Asthma Numeracy Questionnaire		4 items	Validated for use with adults	.57^a^	Question-specific; free text or list of possible answers	0-4	Parents
	Wechsler Individual Achievement Test-III		Varies by grade	Validated for use based on current grade	>.08	—^b^	Varies by grade	Youth
**Asthma control**								
	**Asthma Control Test**		—	Validated for use with ages 4-11 years and >12	—	Version and question specific, for example, 4-point Likert scale from very bad to very good	—	—
	Age 4-11 years version		7 items	—	.76	—	0-27	Parent and youth
	Age >12 years version		5 items	—	.84	—	5-25	Youth

^a^For measures with less than 10 items, an alpha value greater than .05 indicates satisfactory reliability.

^b^—: not applicable.

#### Literacy Assessment

##### Short Test of Functional Health Literacy in Adults

The S-TOFHLA [32] is a measure of a respondent’s ability to read and understand health-related information. Total raw scores <16 are reflective of *inadequate* literacy. The measure will only be administered to parents and participants aged 13 to 17 years, as the measure has been validated in this age group but not with younger children [29].

##### Asthma Numeracy Questionnaire

The ANQ [33] is a validated measure assessing understanding of numerical concepts including risk and percentages in asthma self-management instructions. This 4-item measure will be completed by parents at baseline.

##### Wechsler Individual Achievement Test-III Reading Comprehension Subtest

The Wechsler Individual Achievement Test-III [34]—Reading Comprehension Subtest is a measure of reading comprehension that is widely validated for use in children and adolescents. It will be used as a proxy for health literacy with the full sample of young people in this study because there are no comprehension-based health literacy measures for youth aged less than 13 years. Participants’ raw score on the WIAT-III will be converted to age-based and grade-based standard scores for data analyses, and age-based and grade-based percentile rank.

#### Asthma Symptom Control

The ACT [35] is a measure of asthma health status, including asthma symptom activity. Both versions of the ACT (aged 4-11 years and >12 years) use the same cutoff score of 19, with lower scores indicating problems with asthma symptoms. An average item score will be computed for statistical analyses. Total scores will be reported for descriptive purposes only.

#### Asthma Action Plan Knowledge

A structured interview *(AAP Knowledge Interview*) was designed for this study and will assess participants’ and parents’ understanding of prescribed AAPs at 3 time points, face-to-face (baseline and 3 and 6 months). Two versions of this interview have been created (parent and participant), with 3 parallel forms of each version (A, B, and C) to reduce practice effects across assessments. Respondents can refer to their AAP to answer questions. The first 7 items describe different scenarios involving asthma symptoms commonly experienced by young people, and respondents are asked to identify the relevant zone for each scenario. Each of these items are scored for accuracy (1=correct, 0=incorrect). The final 3 items ask about details of the child’s green, yellow, and red zone instructions. Responses to these final 3 questions are coded for accuracy using the following categories: 2=correct, 1=correct but vague, 0=incorrect. A total of 3 scores are calculated: (1) zone identification, (2) treatment plan knowledge, and (3) total score.

#### Asthma Action Plan Satisfaction

Devised for this study, the *AAP Satisfaction Interview* consists of parallel forms (parent and young person), each consisting of 11 items to assess the perception of the content, clarity, appeal, and utility of AAPs. For example, “How clearly does your (your child’s) asthma action plan explain medicines that you (your child) need(s) to take every day?”. Respondents will answer items using 4-point Likert scales relevant to the wording of each item (eg, *Very helpful* to *Not helpful at all* or *Very clear* to *Not clear at all*), and items are summed to yield a total score. After each question, respondents will be invited to share feedback or experiences related to that question.

#### Pulmonary Function

Pulmonary function will be assessed using spirometry, administered by a trained member of the research team during clinic-based appointments. After maximal inhalation, spirometry measures the volume of air exhaled during a forceful and complete exhalation, as well as the flow of air at different time points. The 4 primary endpoints derived and focused on for the purposes of this study will be (1) the total exhaled volume known as forced vital capacity (FVC; both absolute and percentage of predicted values), (2) the volume exhaled in the first second known as forced expiratory volume in 1 second (FEV1; both absolute and percentage of predicted values), (3) their ratio (FEV1/FVC), and (4) the forced expiratory flow between 25% and 75% of the forced vital capacity (FEF 25-75; percentage of predicted values), which may provide information regarding the small airways.

#### Medication Adherence

Objective adherence data will be collected using an electronic sensor provided by Propeller Health (Madison, WI), a company specializing in mobile self-management technology for respiratory conditions, including asthma. The Propeller Health sensor provides a reliable and objective record of each actuated dose from participants’ inhaler [36,37]. During enrollment, the participant’s treatment plan information will be entered into the Propeller Health online dashboard, including the type of daily controller medication, number of puffs per administration (eg, 1 or 2 puffs), frequency of administrations (eg, once or twice per day), and approximate times per day when they expect to use the inhaler. Data from the Propeller Health sensor will be stored on the device and automatically uploaded to the study’s secure dashboard via a mobile phone app or hub, as previously described. The dashboard can only be viewed by research and Propeller Health team members. Participants will not be able to access their adherence data.

The sensor contains a battery that can last up to 18 months and can hold up to 1000 pieces of data. Therefore, if the sensor and app or hub through which it communicates with the Propeller Health dashboard are not in close proximity for several days, a sync between the sensor and app or hub will effectively bring the adherence data up to date. As a result, time away from home for participants (eg, during vacations or intermittent issues with Wi-Fi connection) will not detrimentally affect the accurate collection of adherence data. Once it is time to replace or refill their inhaler prescription, the family will transfer the sensor to the participant’s next daily controller inhaler. This transfer can occur without any disruption to the collection of adherence data. Before each study visit, the adherence monitoring dashboard will be checked, and the family will be asked to sync their sensor with their smartphone app or hub if a sync has not occurred in recent days.

Families will be asked to have their child use only the inhaler with the device attached for the duration of the study and that this device will track particular aspects of medication use. Reactive effects, if they occur, should be equally distributed across groups. The outcome of mean daily adherence will be calculated as the total number of puffs actuated divided by the total number of puffs prescribed and multiplied by 100. Episodes of 10 or more actuations in less than a minute will be classified as *dumps* (ie, participant’s intentional attempt to appear more adherent) or device error; such data will be excluded from the analyses [38].

### Feasibility and Acceptability Assessment

To assess the feasibility and acceptability of this pilot RCT, qualitative data will be collected by reviewing study records, through interviews with service providers involved in the study and via exit interviews with a subgroup of participants and parents. Interviews will be conducted with providers involved in the study at the beginning and toward the end of the course of the pilot RCT, either face-to-face or by telephone. Finally, semistructured interviews will be conducted with a subgroup of participants and parents following completion of their involvement in the study. Exit interviews will be conducted either face-to-face or by telephone, depending on the availability and preferences of the interviewees.

The interviews will be conducted by a member of the research team who is not involved with the day-to-day running of the study to facilitate openness among the families and providers to share their study experiences and feedback. Table 4 summarizes the feasibility- and acceptability-related variables that will be examined in this study, the data collection method that will be used to gather information related to each variable, and the schedule for data collection.

**Table 4:** Feasibility and acceptability outcomes and data collection plan.

Variable		Data collection method	Schedule
**Feasibility: Is a definitive trial possible? Is it possible to integrate PAAP^a^** **within routine care?**			
	Provider engagement (eg, facilitating and participating in study meetings)	Study records and provider interviews	Ongoing and 3-month intervals
	Recruitment and retention rates	Study records and interviews with providers and families	Ongoing and 3-month intervals
	Fit of study activities within clinic workflow	Provider interviews	3-month intervals
	Integrity of data collection (eg, missing data)	Study records	Ongoing
	Family views of study activities (eg, schedule, time involved, and types of measures)	Interviews with families	Exit interview
	Adequacy of participant payments	Interviews with families	Exit interview
**Acceptability: Does a PAAP meet the needs of physicians and families?**			
	Perceptions of value added to consultations and asthma management	Interviews with providers and families	Providers: 3-month intervals; Families: 1-month phone interview and exit interview
	Adequacy of PAAP (eg, accessibility, clarity, usability, tailoring, liking, and influence)	Interviews with providers and families	Providers: 3-month intervals; Families: exit interview
	Impact of group assignment on retention	Interviews with providers and families	Providers: 3-month intervals; Families: exit interview

^a^PAAP: pictorial asthma action plan.

### Statistical Analyses

#### Power Analysis

The aims of the primary quantitative analyses in this pilot RCT are to assess initial evidence for the efficacy of PAAPs in comparison with WAAPs for improving pediatric asthma management and outcomes and to produce robust effect size estimates ahead of a future definitive RCT. A power analysis was run for the primary outcome of the ACT using PASS software version 13 (NCSS, 2019) [39]. Effects were estimated based on a previous study [40], using WAAP compared with verbal instruction. Power in this study was calculated for effect sizes between 0.50 and 1 SD unit increase in the effect size of the outcome in the treatment group. The group by time interaction effect (σ=2.21, effect size σm/σ ranged from 0.226 to 0.452) was tested in this power calculation, with alpha set to .05. In this study, equivalence tests of means (using 2 one-sided tests on data from a parallel-group design with sample sizes of 25 in the WAAP group and 25 in the PAAP group) will result in 82% power at a 5.0% significance level when the true difference between the means is 0.0, the SD is 3.5, and the equivalence limits are −3.0 and 3.0. Therefore, results that fall within an SD unit difference would indicate that PAAP is *as good as* guideline-recommended care (WAAP) with sufficient power.

On the basis of our previous research of similar duration, we anticipate a<20% attrition rate. Thus, we will recruit up to 60 participants from the specialist asthma and pulmonology clinics within WVU Medicine, with sufficient power to test for equivalence even with attrition and not accounting for missing data. In the event of a lower-than-anticipated attrition rate or data missing completely at random, a sample size of 50 (25 per group) will maintain high power for detecting equivalence.

#### Quantitative Data

Preliminary evidence of between-group differences will be examined in relation to the following outcome variables: AAP knowledge, AAP satisfaction, ACT scores, mean adherence to daily controller medication, and pulmonary function using spirometry. The hypothesis tests of interest will involve the fixed effects of the time-by-group interaction for all study outcomes, with covariates included provided they are significant and improve model fit. Specifically, to account for the correlation among the repeated measurements on each participant, general linear mixed models will be the main tools of analysis for the primary outcomes. These models are designed to model correlations among observations on subjects (over time and within groups) and are valid in the presence of missing at random (MAR) data [41]. Various random effects and covariance estimates will be compared using the Akaike Information Criterion to determine the best fitting model. Random effects, considering the individual level (rather than just population) parameters, will be included and may also be examined in the event of assessing best treatment practices. Accurate model parameter estimation will be ensured by the use of the residual maximum likelihood (REML) approach to estimation (appropriate for use with MAR missing data), and the Kenward-Roger approximation of the degrees of freedom will allow for accurate inference.

Given our power to estimate effect sizes for a subsequent definitive RCT, we will demonstrate the overall stability of the effect of the treatment response utilizing the *F* test for equivalence of the effect of psi-squared, a standardized measure of difference between groups/time point means and an overall (across groups and time points) mean. This will be assessed utilizing output from the best-fitting general linear mixed model and by testing whether this psi-squared effect size measure falls in the critical region, following the method outlined by Wang and Amrhein [42].

SPSS version 24 will be used for data management and basic analyses; SAS version 9.4 (primarily PROC MIXED) will be used for all advanced statistical analyses. Every attempt will be made to minimize attrition and missing data; however, we recognize that some degree of missing data is inevitable. The REML method involved in mixed modeling is generally appropriate for use under the MAR assumption. Moreover, we will carry out models under missing not at random assumptions to assess the sensitivity of our conclusions to the missing data (eg, via selection models or pattern-mixture models, as needed or appropriate).

#### Qualitative Data

Thematic analysis will be used to organize and analyze the qualitative data gathered from providers, participants, and parents, including AAP satisfaction survey and exit interview data [43]. The aim of thematic analysis is to facilitate the identification of patterns of meaning across a qualitative dataset. Thematic analysis can be used to address a wide range of research questions and is widely used, increasingly as part of the processes of development, pilot testing, and evaluation of behavioral trials [43,44]. Data from interviews with providers and exit interviews with participants and parents will be transcribed verbatim and entered into *Atlas.ti* software for analysis. Data from the AAP satisfaction survey (1- and 6-month follow-up) will be transcribed and entered into a Microsoft Excel database for analysis.

In this study, a theoretical thematic analysis will be conducted with the aim of addressing the research questions related to the feasibility and acceptability of the TAAC intervention and study procedures, as opposed to approaching the data with a more exploratory aim. Analysis will progress through the phases recommended by Braun and Clarke [43], including cycles of repeated reading of transcripts, assigning descriptive codes to the data, categorizing codes under representative themes, defining and editing themes, and presenting the report of findings. All data from provider and exit interviews will be coded by one member of the research team (LH). A research assistant will independently code 20% of the provider and exit interview data, and agreement between coders will be assessed and discussed. Data from the AAP satisfaction structured interviews will be analyzed by a research assistant, once a coding framework is agreed upon between 2 coders (LH and research assistant).

## Results

Recruitment and data collection began in May 2017 and were completed in October 2018. Results are expected by March 2019.

## Discussion

According to the literature, many barriers exist to asthma self-management among young people, including inadequate asthma education and knowledge [13]. Although WAAPs are an evidence-based intervention [3] designed to address these concerns, many people with asthma are not receiving or utilizing WAAPs [45] despite national guideline directives [3]. Barriers to effective asthma self-management may be addressed by the introduction of more accessible and personalized PAAPs. This study aims to assess the feasibility and acceptability of the TAAC intervention and study procedures, for example, the usability of the PAAP software and appeal of the PAAPs generated by the software. We also aim to assess whether literacy and health literacy among children and adolescents and health literacy in caregivers are associated with asthma knowledge and health outcomes. We hypothesize that relative to WAAPs, PAAPs will produce significantly greater improvements in asthma care knowledge of children and adolescents and their caregivers, their satisfaction with education regarding the child’s asthma care and subsequent self-efficacy for managing symptoms accordingly, asthma health outcomes (ie, asthma symptom control and pulmonary function), and adherence to the prescribed medical regimen. It is also hypothesized that these improvements will occur as a function of PAAPs having preferred features, such as simplified and reduced text, lower literacy demand, appealing appearance, and accessible format and content.

### Strengths and Limitations

Notable strengths of this protocol include the mixed-methods approach to data collection and analysis; integration of technology in the intervention; use of objective outcomes measures; identification of BCTs; and the approach of adapting the standard WAAP, an existing guideline-based tool. The findings of this study will inform the design of the next phase of this research, which will be a definitive RCT. Potential limitations of the protocol are mainly a function of the pilot nature of the study, including data collection from a small number of sites, implementation of the intervention by a small number of physicians, and reliance on Wi-Fi in a rural location in the United States. Nevertheless, this study will produce valuable pilot data for a future large-scale definitive trial that will aim to recruit a large sample from multiple sites and have substantial potential for national application.

### Future Research

The planned feasibility and pilot trial, as part of a larger program of research, builds on a growing body of innovative AAP literature [19-23]. In particular, patient education materials using a largely pictorial format may also be applicable to people with other complex conditions, such as type 1 diabetes and cystic fibrosis. Our future research will include the development of pictorial tools for diverse populations, including people with a first language other than English. We will draw on implementation science frameworks to design and conduct this research with a view to the adoption of the use of PAAPs in routine practice.

## Abbreviations

AAP: asthma action plan
ACT: Asthma Control Test
ANQ: Asthma Numeracy Questionnaire
BCT: behavior change techniques
EMR: electronic medical record
FEF 25-75: forced expiratory flow between 25% and 75% of the forced vital capacity
FEV1: forced expiratory volume in 1 second
FVC: Forced Vital Capacity
HHS: Health and Human Services
HRSA: Health Resources and Services Administration
MAR: missing at random
PAAP: pictorial asthma action plan
RCT: randomized controlled trial
REML: residual maximum likelihood
S-TOFHLA: Short Test of Functional Health Literacy in Adults
TAAC: Take Action for Asthma Control
WAAP: written asthma action plan
WIAT-III: Wechsler Individual Achievement Test-III
WVU: West Virginia University
